# Can the simplified magnetic resonance index of activity be used to evaluate the degree of activity in Crohn's disease?

**DOI:** 10.1186/s12876-021-01987-z

**Published:** 2021-10-28

**Authors:** Yongli Tao, Hong Li, Han Xu, Wen Tang, Guohua Fan, Xiaochun Yang

**Affiliations:** 1 Department of Radiology, The Second Affiliated Hospital of Soochow University, Suzhou, 215004 Jang Su China; 2 Department of Gastroenterology, The Second Affiliated Hospital of Soochow University, Suzhou, Jang Su, China

**Keywords:** Crohn’s disease, MaRIAs, Inflammatory indicators, SES-CD, Activity

## Abstract

**Background and aims:**

A simplified magnetic resonance index of activity (MaRIAs) was recently proposed. Our aim was to verify whether MaRIAs can accurately assess the activity degree of CD.

**Methods:**

We retrospectively analyzed the MRI, ileocolonoscopy, fecal calprotectin (FC), erythrocyte sedimentation rate (ESR) and C-reactive protein (CRP) data of 93 CD patients. With the SES-CD as the gold standard, MaRIAs’ accuracy, the correlation of MaRIAs and SES-CD, FC, ESR, CRP, and interevaluator reliability were assessed.

**Results:**

MaRIAs ≥ 1 detected segments with active CD with 90.80% specificity and 81.37% sensitivity (area under the curve was 0.91, 95% confidence interval 0.87–0.94). MaRIAs score of 2 or more detected severe lesions with 88.89% specificity and 95.12% sensitivity (AUC was 0.96, 95% confidence interval was 0.94–0.98). The MaRIAs score showed a high correlation with the SES-CD in the terminal ileum, transverse colon, right colon, and left colon (*r* = 0.85, 0.91, 0.88, 0.86, *P* < 0.001) and a moderate correlation with the SES-CD in the rectum (*r* = 0.74, *P* < 0.001). The global MaRIAs score was highly correlated with the global SES-CD (*r* = 0.90, *P* < 0.001). The global MaRIAs score was positively correlated with the fecal calprotectin (FC), erythrocyte sedimentation rate (ESR), and C-reactive protein (CRP) (*r* = 0.77, *r* = 0.64, and *r* = 0.68). The intragroup correlation coefficient (ICC) of the two physicians was nice in the terminal ileum, the right colon, the transverse colon, the left colon and was moderately good in the rectum.

**Conclusion:**

MaRIAs can accurately evaluate the disease activity level of CD and are highly correlated with SES-CD and biomarkers. The interrater reliability of the two physicians was moderately good to nice.

## Introduction

Crohn's disease (CD) is a chronic transmural inflammatory bowel disease of unknown origin that can involve any part of the digestive tract, especially the terminal ileum and right colon, with symptoms such as abdominal pain, diarrhea, abdominal mass, and perianal fistula. The initial symptoms of CD are not obvious, and remission and recurrence occur alternately, with multiple internal and external complications. The incidence has been increasing in recent years [1]. The poor efficacy of drugs and the high disability rate make the diagnosis and treatment very challenging.

Endoscopy is currently considered the gold standard for the diagnosis of CD. The simple endoscopic score for Crohn's disease (SES-CD), including ulcer size, ulcer area, lesion area and intestinal stenosis, can accurately evaluate the disease and is simple to calculate, so it is the most widely used endoscopic scoring system [2, 3]. However, extraintestinal conditions cannot be assessed with endoscopy; this procedure is invasive and not suitable for patients with intestinal stenosis, and other limitations have been described in a number of studies[4–6].

Magnetic resonance imaging (MRI) has good soft tissue resolution and does not involve ionizing radiation and is noninvasive. It can be used to observe the whole abdomen pelvic cavity and to evaluate disease activity, mesenteric blood vessels and lymph nodes, and disease-related complications in patients with CD and is particularly attractive because healing of the mucosa and deeper layers of the bowel wall can be assessed [7]. Therefore, it is widely used in the diagnosis and long-term follow-up of CD. The magnetic resonance index of activity (MaRIA) [8] is currently the most widely used and studied MRI scoring system for CD [9–11].

However, there are some limitations, such as its complicated calculation and the large selection error of the region of interest (ROI), for patients with thin intestinal walls. In March 2019, the Rimola [12] team proposed a simplified magnetic resonance index of activity (MaRIAs) for CD.

To better assist clinical work and promote the application of MR in CD, this study took the SES-CD as the gold standard and retrospectively analyzed the ability of the MaRIAs to detect the activity degree of CD patients and the correlation between the MaRIAs score and clinical inflammatory indicators to explore the effectiveness of the MaRIAs score in evaluating the degree of CD activity.

## Methods

### Patients

This was a retrospective study of 107 patients with CD who were treated at the Department of Gastroenterology of the Second Affiliated Hospital of Soochow University between March 2017 and September 2019. Within one week, an MR examination and ileocolonoscopy were performed, and the fecal calprotectin (FC), erythrocyte sedimentation rate (ESR) and C-reactive protein (CRP) were determined.

The exclusion criteria were as follows: 1) poor quality of endoscopic or magnetic resonance images; 2) incomplete clinical data; 3) history of taking nonsteroidal anti-inflammatory drugs (NSAIDs) within one week before the FC test; and 4) other intestinal lesions. Of a total of 107 CD patients, 14 were excluded: 3 for failed ileocolonoscopy because of severe strictures, 2 for poor-quality MR images, 6 for incomplete clinical data, and 3 for NSAID use. In all, 93 patients were included. The project was approved by the ethics committee of the Second Affiliated Hospital of Soochow University. Because of the retrospective nature of the study, the need for individual consent was waived.

### MR examination

The patient fasted for 8 h before the MR examination and took an oral 2000 ml of 4% mannitol aqueous solution to fill the intestine (500 ml every 15 min) 1 h before the examination. To inhibit bowel peristalsis, intramuscular injection of 10 mg of choline was given 10 min prior to examination.

A Philips Ingenia 3.0 T magnetic resonance scanner and abdominal phased array coil were used for examination. The patient was placed in the supine position, and the scanning sequence was moved from the head side to the foot side. The conventional MRE scanning sequence and parameters were as follows: (1) BFFE-BH-COR: TR: 3.0 ms, TE: 1.52 ms, terms slice thickness: 5 mm, slice gap: 0 mm, flip angle: 40°, and matrix: 268 × 206; (2) T2WI-TSE-COR: TR: 1100 ms, TE: 80 ms, terms slice thickness: 5 mm, slice gap: 0 mm, flip angle: 90°, and matrix: 376 × 290; (3) T2WI-SPAIR-COR: TR: 869 ms, TE: 80 ms, terms slice thickness: 5 mm, slice gap: 0 mm, flip angle: 90°, and matrix: 280 × 251; (4) T2WI-SPAIR-TRA: TR: 869 ms, TE: 80 ms, terms slice thickness: 5 mm, slice gap: 0 mm, flip angle: 90°, and matrix: 232 × 262; (5) mDIXON-W-BH: TR: 3.8 ms, TE1: 1.32 ms, TE2: 2.4 ms, terms slice thickness: 5 mm, slice gap: 0 mm, flip angle: 10°, and matrix: 252 × 151; (6) transverse diffusion weight imaging (DWI): b values of 0, 300, 600, and 1000 s/mm^2^, TR: 860 ms, TE: 64 ms, terms slice thickness: 5 mm, slice gap: 0.5 mm, flip angle: 90°, and matrix: 132 × 135; and (7) after the IV administration of 0.2 ml/kg of gadolinium chelate(omniscan, 0.5 mmol/ml) at an injection rate of 2–3 ml/s, dynamic images including precontrast, arterial, portal venous and equilibrium phase images were acquired in the coronal plane: TR: 1.32 ms, TE: 3.7 ms, terms slice thickness: 5 mm, slice gap: -2 mm, flip angle: 10°, and matrix: 268 × 235.

### Endoscopy and laboratory testing

Each patient consumed liquid food the day before colonoscopy and fasted for 8 h before the examination. Three bags of compound polyethylene glycol electrolyte were dissolved in 3000 ml of warm water and orally administered 4 h before the test until the feces were clear. A gastroenterologist with more than 5 years of endoscopy experience performed endoscopic examination of CD patients and reported CD lesions according to the SES-CD. The bowel segments was divided into 5 segments: 1) the terminal ileum (the ileum that can be reached by endoscopy); 2) the right colon (ileocecal, cecum, and ascending colon); 3) the transverse colon; 4) the left colon (descending colon and sigmoid colon); and 5) the rectum. For each intestinal segment, an SES-CD between 0 and 2 is considered as indicated remission, 3–6 as mild disease, and ≥ 7 as moderate to severe disease[3, 14]. In addition, a classification of severity on a segment basis was performed by considering the presence of severe lesions (ulcers with a diameter > 5 mm) [15]. The global SES-CD is the sum of the SES-CDs of each intestinal segment: 0–3 indicated remission, 4–10 mild disease, 11–19 moderate disease and ≥ 20 severe disease. Therefore, in this study, patients were considered to have active disease if the global SES-CD was ≥ 4 and severe disease if the global SES-CD was ≥ 20 [16].

The laboratory indexes included the FC, ESR and CRP level. The FC level was measured by quantitative enzyme-linked immunosorbent assay (ELISA). The FC level was determined one day before colonoscopy. The normal range of FC was < 200 µg/g. The ESR and CRP level were determined according to standard laboratory procedures. The normal range of CRP was 0–5 mg/L, and the normal range of the ESR was 0–20 mm/h. Using the global SES-CD as the gold standard, the patients were divided into remission group, mild to moderate activity group and severe activity group, and the laboratory examination results of different activity levels are shown in Tables 1, 2, 3.

**Table 1:** Clinical features of all the patients

Variables	
Male, n (%)	63 (68)
Age, years; median (IQR)	29 (25–37)
Disease duration, years; median (IQR)	3 (1–6)
Montreal classification [13]	
*Age at diagnosis (years), n (%)*	
A1 (under 16)	2 (2.15)
A2 (17–40)	74 (79.57)
A3 (over 40)	17 (18.28)
*Disease location, n (%)*	
L1 (terminal ileum)/(*L4)	15 (16.13)/9 (9.68)
L2 (colon)/(*L4)	12 (12.90)/2(2.15)
L3 (ileum plus colon)/(*L4)	66 (70.97)/37 (39.78)
*Disease behavior, n (%)*	
B1 (nonstricturing, nonpenetrating)	49 (52.69)
B2 (stricturing)	12 (12.90)
B3 (penetrating)	32 (34.41)
Perianal involvement, n (%)	37 (39.78)
*Surgical history, n (%)*	
History of perianal surgery	28 (30.11)
History of partial bowel resection	3 (3.23)
*Treatment, n (%)*	
No treatment	15 (16.13)
Steroids	10 (10.75)
Immunomodulator	31 (33.33)
Anti-TNF inhibitor	37 (39.78)
Abdominal abscess, n (%)	3 (3.23)
Intestinal fistula, n (%)	5 (5.38)
Colovesical fistula, n (%)	1 (1.08)
Ileal bladder fistula, n (%)	1 (1.08)

IQR: interquartile range; *L4: L1/L2/L3 with upper gastrointestinal disease

**Table 2:** Statistical results of the activity degree of each intestinal segment

Intestinal segments	Remission	Mild to moderate active	Severe disease
Terminal ileum	26 (28%)	54(58%)	13 (14%)
Right colon	66 (71%)	19 (20%)	8 (9%)
Transverse colon	64 (69%)	22 (23%)	7 (8%)
Descending colon and sigmoid colon	56 (60%)	25 (27%)	12 (13%)
Rectum	49 (53%)	38 (41%)	6 (6%)
Total	261 (56%)	158 (34%)	46 (10%)

**Table 3:** Laboratory examination results of different activity levels on ileocolonoscopy per patient

Biomarkers, median (IQR)	Remission (0–3)	Mild to moderate active (4–19)	Severe active (≥ 20)	*P* value
FC (µg/g)	118 (42–275.25)	589 (267–1369)	1800 (1791.75–1800)	< 0.001
CRP (mg/L)	5.6 (5.15–5.6)	6.5 (5.6–24)	87.65 (54–94.75)	< 0.001
ESR (mm/h)	4 (2–8)	19 (7–46)	66.5 (53.25–88.5)	< 0.001

IQR: interquartile range; FC: fecal calprotectin; CRP: C-reactive protein; ESR: erythrocyte sedimentation rate

### MR image analysis

**Fig. 1:**
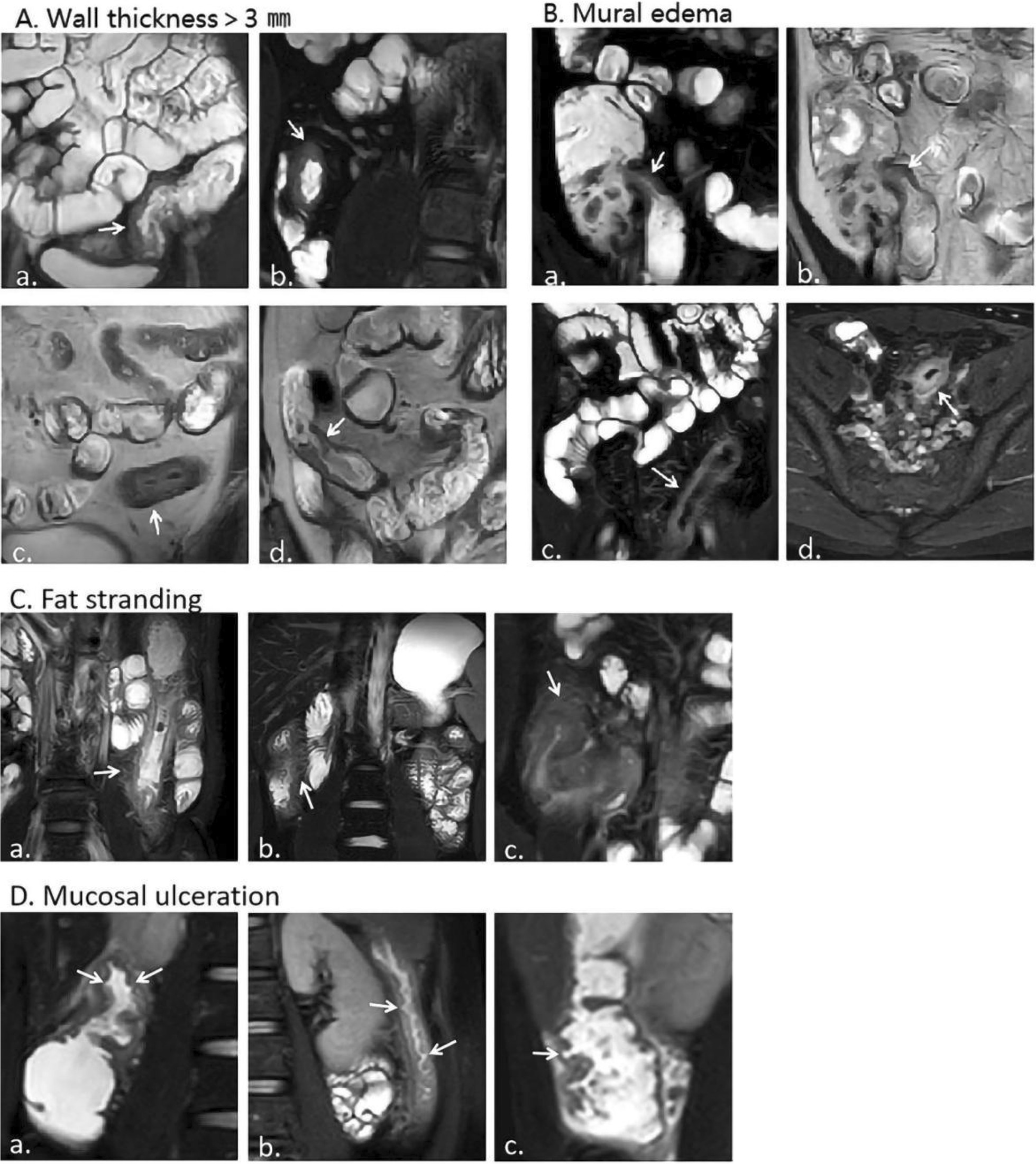
Representative examples of magnetic resonance (MR) lesions: **a** Wall thickness > 3 mm: coronal T2-weighted images with fat saturation of the descending colon (a) and terminal ileum (b); coronal T2-weighted images of the descending colon (c) and terminal ileum (d) (arrow in image). **b** Mural edema (high signal intensity on T2 sequences with fat saturation compared with normal appearing loops): coronal T2-weighted images with fat saturation (a) and without (b) of the terminal ileum from the same patient show high signal intensity. Coronal (c) and axial (d) T2-weighted images with fat saturation of the descending colon from the same patient (arrow in image). **c** Fat stranding (loss of the normal sharp interface between the intestinal wall and mesentery, with edema/fluid in the perienteric fat): coronal T2-weighted images with fat saturation of the descending colon (a), and ascending colon (b, c) (arrow in image). **d** Mucosal ulceration: coronal T2-weighted images with fat saturation of the ascending colon (a, c) and descending colon (b) (arrow in image)

The MR segmentation method is the same as the endoscopic segmentation method. The MaRIAs score in each segment was calculated by the following formula: = wall thickness (> 3 mm) × 1 + edema × 1 + fat stranding × 1 + ulcers × 2 (Fig. 1). Two radiologists with more than 5 years of experience in MR abdominal readings scored the MR images separately under the premise that the results of the colonoscopy and laboratory examination were unknown. For the four variables, when the evaluation results of the two experts were inconsistent, the final answer was determined after discussion. The scores of each intestinal segment were determined separately. MaRIAs score ≥ 1 indicated intestinal segment activity, and MaRIAs score ≥ 2 indicated severe activity. The global MaRIAs score is the sum of the MaRIAs scores of each intestinal segment.

### Statistical analysis

SPSS 25.0 statistical software was used for data analysis. The intraclass correlation coefficient was used to evaluate the consistency between two physicians. The measurement data were normalized by the Shapiro–Wilk method; the mean ± standard deviation is reported for normally distributed data, and the median (interquartile range) is reported for data that did not satisfy a normal distribution. The Kruskal–Wallis test was used to compare the variables among the three groups, and the Bonferroni method was used for correction. A *p* value of < 0.05 was considered statistically significant. Receiver operating characteristic curves (ROC) were drawn to evaluate the effectiveness of the MaRIAs for assessing CD activity. Correlations between the MaRIAs score and the SES-CD, FC, CRP, and ESR were measured by Spearman correlation tests.

## Results

### Endoscopic evaluation results

A total of 93 patients with 465 intestinal segments were included in this study, including 93 segments of the terminal ileum and 372 segments of the colorectum. Their basic information is shown in Table 1. Three patients had a history of intestinal resection (two partial small bowel resections and one ileocecal resection), which did not affect observation or analysis. SES-CD of the intestinal segments indicated that 261 (56%) segments were associated with remission, 158 (34%) with mild to moderate disease, and 46 (10%) with severe disease; ulcers were found in 46 of the segments (Table 2). 14 (15%) patients were in remission, 61 (66%) patients had mild to moderate disease and 18 (19%) patients had severe disease, with the global SES-CD as the gold standard.

### Laboratory evaluation results

The results of the laboratory examination are shown in Table 3. With an increase in CD activity, each index increased to different degrees, and the difference was statistically significant.

### MR imaging evaluation results

#### Scoring results for the MaRIAs

The occurrence ratio of the 4 MR variables at different levels of activity is shown in Table 4. Ulcers and fat stranding appeared in only one case in remission group, the incidence of ulcers and fat stranding in severe active group was significantly increased compared with remission group and mild-moderate active group, and the difference between the three groups was statistically significant. The median global MaRIAs for those patients in remission or for those with mild to moderate or severe disease were 0, 3 (1–5) and 11 (8.25–12.75), respectively. The higher the degree of activity was, the higher the MaRIAs score. The difference was statistically significant (*P* < 0.001).

**Table 4:** MR variables results of the intestinal segments with different activity levels

Variables	Remission	Mild to moderate active	Severe disease	*P* value
Wall thickness	50 (19%)	110 (54%)	46 (100%)	< 0.001
Edema	40 (15%)	125 (61%)	46(100%)	< 0.001
Fat stranding	1 (0.4%)	17 (8%)	27 (59%)	< 0.001
Ulcers	1 (0.4%)	18 (9%)	38 (83%)	< 0.001

The details about the presence of the 4 MR variables across the different levels of disease activity are shown in Table 5. When judging whether there was activity in the intestinal segment, wall thickness > 3 mm and edema showed good diagnostic efficiency. The specificity of fat stranding and ulcers in judging intestinal activity is as high as 99%. The diagnostic efficiency of edema, fat stranding, and ulcers in diagnosing serious intestinal segment activity was successively improved (AUC: 0.60, 0.75, 0.82). The sensitivity of edema in diagnosing severe intestinal segment activity is as high as 100%, but the specificity is very low. Wall thickness was not statistically significant in distinguishing between mild to moderate and severe disease periods (*P* = 0.2).

**Table 5:** Diagnostic accuracy of 4 MR variables to identify active and severe diseases on ileocolonoscopy per intestinal segment

variables	AUC	95%	Sensitivity(%)	Specificity(%)	*P* Value
*Active*					
Wall thickness	0.83	0.80–0.87	88.24	78.54	< 0.0001
Edema	0.85	0.81–0.89	84.31	86.21	< 0.0001
Fat stranding	0.60	0.54–0.66	22.06	99.23	< 0.0001
Ulcers	0.69	0.63–0.73	37.75	99.62	< 0.0001
*Severe disease*					
Edema	0.60	0.51–0.68	100	20.25	0.043
Fat stranding	0.75	0.66–0.84	60.87	89.24	< 0.0001
Ulcers	0.82	0.75–0.89	86.96	76.58	< 0.0001

#### The efficacy of MaRIAs in assessing the activity of patients with CD

**Fig. 2:**
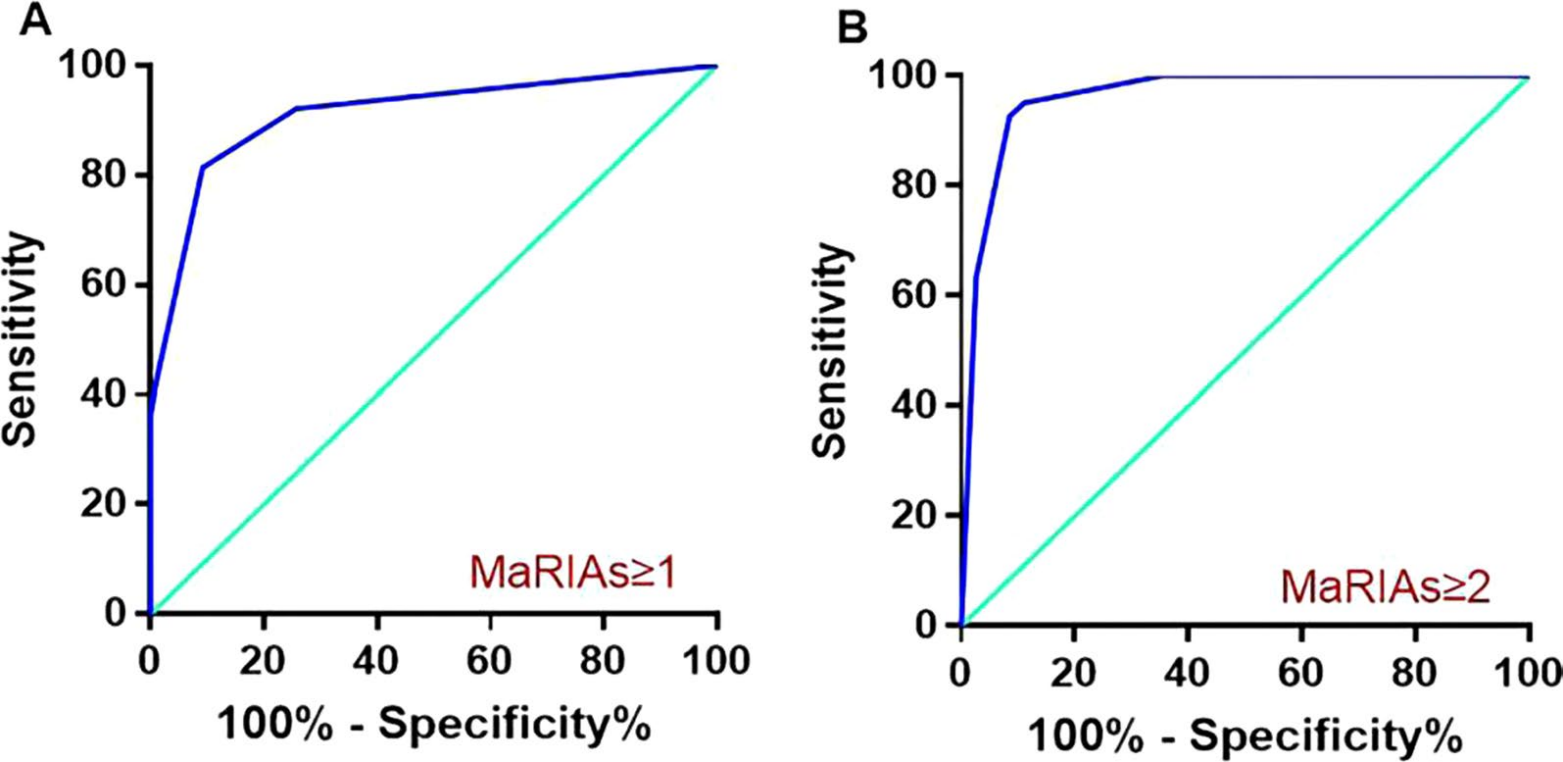
The ROC curve prediction of disease activity (**a**) and severe activity (**b**) associated with each segment showed that the MaRIAs could accurately assess the degree of CD activity

MaRIAs ≥ 1 detected segments associated with active CD with 90.8% specificity and 81.37% sensitivity (area under the curve (AUC) was 0.91, 95% confidence interval was 0.87–0.94). MaRIAs score of 2 or more detected severe lesions with 88.89% specificity and 95.12% sensitivity (AUC value was 0.96, 95% confidence interval was 0.94–0.98) (Fig. 2). The diagnostic accuracy of the MaRIAs based on separate subanalyses for each intestinal segment is presented in Tables 6 and 7.

**Table 6:** Diagnostic accuracy of MaRIAs ≥ 1 for the identification of active disease on ileocolonoscopy per intestinal segment

Intestinal segments	AUC	Sensitivity (%)	Specificity (%)	*P* Value
Terminal ileum	0.97	95.52	92.31	< 0.001
Right colon	0.87	77.78	90.91	< 0.001
Transverse colon	0.93	89.66	92.19	< 0.001
Descending colon and sigmoid colon	0.92	86.49	82.14	< 0.001
Rectum	0.79	86.36	53.06	< 0.001
Total	0.91	81.37	90.8	< 0.001

**Table 7:** Diagnostic accuracy of MaRIAs ≥ 2 for the identification of severe disease on ileocolonoscopy per intestinal segment

Intestinal segments	AUC	Sensitivity (%)	Specificity (%)	*P* Value
Terminal ileum	0.96	100	78.41	< 0.001
Right colon	0.98	100	89.41	< 0.001
Transverse colon	0.97	100	74.7	< 0.001
Descending colon and sigmoid colon	0.97	73.54	87.65	< 0.001
Rectum	0.98	100	86.21	< 0.001
Total	0.96	95.12	88.89	< 0.001

#### Correlation analysis between the MaRIAs score and SES-CD

**Fig. 3:**
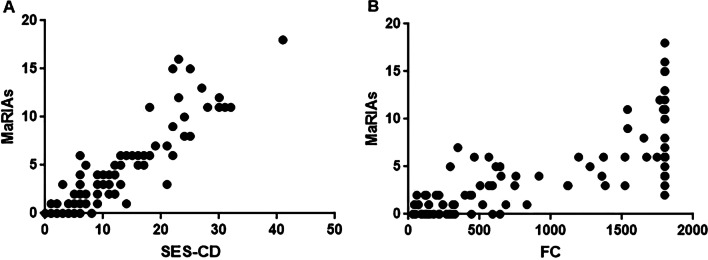
Correlation between the MaRIAs score and SES-CD in patients (**a**) and between the MaRIAs score and FC level in patients (**b**)

MaRIAs score and SES-CD were highly correlated in the terminal ileum, right colon, transverse colon, and left colon (*r* = 0.85, *r* = 0.91, *r* = 0.88,* r* = 0.86, *P* < 0.001) and moderately correlated in the rectum (*r* = 0.74, *P* < 0.001). The global MaRIAs score was highly correlated with the global SES-CD (*r* = 0.90, *P* < 0.001) (Fig. 3A).

#### Correlation analysis between the MaRIAs score and laboratory indexes

Correlation analysis showed moderate correlations between the total MaRIAs score and the FC level (Fig. 3B), ESR and CRP level (*r* = 0.77, *r* = 0.64, *r* = 0.68, *P* < 0.001).

#### Interrater reliability assessment

The agreement between the two raters was nice in the terminal ileum, the right colon, the transverse colon, the left colon and was moderately good in the rectum (Table 8).

**Table 8:** Interrater agreement analysis between the two raters

variables per segments	Kappa value	Positive(radiologist 1/2)	Agreement proportion	*P* value
*The terminal ileum*				
Wall thickness	0.84	74/70	86/93	< 0.001
Edema	0.83	68/69	85/93	< 0.001
Fat stranding	0.80	18/18	86/93	< 0.001
Ulcers	0.78	18/22	84/93	< 0.001
*The right colon*				
Wall thickness	0.75	25/22	84/93	< 0.001
Edema	0.71	24/20	83/93	< 0.001
Fat stranding	0.76	7/4	90/93	< 0.001
Ulcers	0.86	12/13	90/93	< 0.001
*The transverse colon*				
Wall thickness	0.80	29/27	85/93	< 0.001
Edema	0.74	24/28	80/93	< 0.001
Fat stranding	0.79	5/5	91/93	< 0.001
Ulcers	0.87	7/9	91/93	< 0.001
*Descending colon and sigmoid colon*				
Wall thickness	0.80	57/58	84/93	< 0.001
Edema	0.72	49/56	80/93	< 0.001
Fat stranding	0.80	16/13	88/93	< 0.001
Ulcers	0.86	21/24	88/93	< 0.001
*Rectum*				
Wall thickness	0.69	50/60	79/93	< 0.001
Edema	0.68	39/45	77/93	< 0.001
Fat stranding	0.64	6/9	88/93	< 0.001
Ulcers	0.60	21/12	82/93	< 0.001

## Discussion

In recent years, with the rapid development of MRI, its application in CD has become increasingly extensive, and it has been recommended for continuous assessment of the disease activity of CD patients [17]. Many methods have been developed to evaluate CD lesions by MR, The most recent evaluation method was proposed by the Rimola team [12] and is the MaRIAs, with the advantages of being simple and convenient. The results of this study show that MaRIAs can accurately assess the degree of activity of Crohn's disease.

In this study, the SES-CD was used as the gold standard to explore the value of the MaRIAs to evaluate the degree of CD activity. The results suggest that as the activity increases, the probability of wall thickness > 3 mm, edema, fat stranding, and ulcers all showed an upward trend, and the total MaRIAs score increases accordingly. This result indicates that the MaRIAs scores can reflect changes in the degree of lesion activity regardless of whether it is in a single intestinal segment or in the total intestine.

Mucosal healing is considered a possible treatment endpoint because it can reduce the hospitalization rate, surgery rate, and corticosteroid use in CD patients [18]. CD is a transmural disease from a pathophysiological standpoint, and achieving mucosal healing may not reflect the ongoing inflammation and intestinal damage occurring beneath the surface of an endoscopically healed lumen [19]. One case of fat stranding in the remission group of this study may have been cured on the mucosal surface of the intestine, but there were lesions on the submucosa and serous surface. MRI can reflect the conditions of the serosal surface of the intestinal wall and the abdominal cavity. An increasing number of studies have shown that MRI can monitor the treatment response. This has led some experts to recommend the use of MRI standards as the treatment endpoint in the clinic.

Adequate patient preparation is a prerequisite for high-quality MRE. A well-filled bowel can more fully show the disease and reduce the probability of missed diagnosis and misdiagnosis. One case of ulcer in remission may be caused by poor intestinal filling and folds of the intestinal wall leading to false positives.

The most important finding in this study was that MaRIAs ≥ 1 and ≥ 2 were the best cut-off values to identify active and severe disease, respectively, which is similar to the results of the Rimola team [12] and Capozzi’ study [20]. They both used the Crohn's Disease Endoscopic Index of Severity (CDEIS) as the gold standard. Compared with the CDEIS, the SES-CD is simpler and highly related to the CDEIS^3^. MaRIAs are highly correlated with the degree of activity under ileocolonoscopy, and the Rimola team [12] found that the MaRIAs were significantly correlated with the CDEIS and MaRIA [12]. Roseira et al. [21] and we all found that MaRIAs were significantly correlated with SES-CD, and we further analyzed the correlation between MaRIAs and CRP and ESR to explore the diagnostic efficacy of MaRIAs.

Another important finding was that when diagnosing intestinal activity, the diagnostic value of edema was the highest. Mural edema is a characteristic feature of active inflammation of the bowel wall and can be detected as an increase in the signal on T2 sequences. Wall thickness > 3 mm is closely related to the presence and severity of the activity [22]. When the lesion is severe, edema can extend to the mesentery. In our study, the sensitivity of edema to diagnose severely active lesions was as high as 100%. CD is a transmural inflammation, and using fat stranding to replace the relative enhancement degree can effectively reflect inflammation. Compared with edema and fat stranding, ulcers are the most effective in diagnosing the presence of severe activity in the intestine. Detailed analysis of the diagnostic performance of each variable on the degree of diseased intestinal activity is the innovation of this study.

Inflammatory indicators can reflect the activity of the disease [23, 24]. FC is an important marker of intestinal inflammation. Compared with the ESR and CRP, FC is not affected by factors outside the intestinal tract and has high specificity [25], which can better reflect intestinal inflammation. The results showed that there was a moderate correlation between the MaRIAs and the FC, ESR and CRP levels. FC is a specific indicator of intestinal inflammation. In this study, the correlation analysis between MaRIAs and biological indicators showed that FC and MaRIAs had the strongest correlation, but they did not show excellent specificity with the MaRIAs, which may be related to the distribution of lesions in the samples and deep ulcers. A number of studies have also shown that the FC level is related to the lesion site [15, 26].

The MaRIA is the most widely used MR scoring system for evaluating the activity and severity of CD. However, the calculation of the relative contrast enhancement is complex and time consuming. Compared with the MaRIA, the MaRIAs have the following three advantages: first, the calculation is simpler and more convenient. A recent study [27] in 121 CD patients confirmed that MaRIAs and MaRIA are highly correlated (r = 0.93; 95% confidence interval: 0.90–0.95). Compared with MaRIA, MaRIAs showed a higher active disease value (area under the curve (AUC): 0.92), and MaRIAs (single intestinal segment) ≥ 2 points was the best cut-off value for predicting the presence of ulcers (AUC: 0.93). Second, the MaRIA score includes the normal intestinal segments, while the MaRIAs score calculates only the diseased segments and can more realistically reflect the pathological changes in the intestine. Third, compared with MaRIA, MaRIAs do not require the use of intravenous contrast, which can shorten the examination time and reduce the examination cost of patients. Without affecting the accuracy, the above advantages make MaRIAs a more favorable tool.

In this experiment, the two radiologists' interrater agreement was moderately good to nice, per variable and per segment, which indicates that the MaRIAs score is stable. The two radiologists' assessment results in the rectum were moderately consistent compared to those in the other segments of the intestine, which may be related to inadequate intestinal expansion [28].

Our study had some limitations. First, this was a single-center study. Second, all treated patients were grouped together, but different treatments might have different impacts on the MRI findings.

However, our study has a few strengths. It had a large sample size and evaluated more than 400 intestinal segments by MR and ileocolonoscopy. The calculations of the confidence interval of ROC analysis and interrater agreement analysis between the two radiologists were performed per segment to fully verify the accuracy and stability of the MaRIAs. Furthermore, we analyzed in detail the occurrence probability of 4 MR variables in the intestinal segments of different activity levels and the diagnostic efficacy of each variable on activity and severe activity. Finally, the correlation between inflammatory factors and the MaRIAs score was analyzed to further verify the reliability that the MaRIAs can be used to analyze the activity degree of CD in this study.

In conclusion, our study demonstrated that MaRIAs can be used to accurately assess the activity degree of CD and were highly correlated with SES-CD, the gold standard, and moderately to well correlated with three inflammatory indicators. Moreover, the interrater agreement analysis between the two radiologists was stable, which demonstrated that the MaRIAs can be applied in the clinic very well.

## Data Availability

The data that support the findings of this study are available from the corresponding author upon reasonable request.

## Abbreviations

CD: Crohn’s disease
MaRIA: Magnetic resonance index of activity
MaRIAs: Simplified magnetic resonance index of activity
SES-CD: Simple endoscopic score for Crohn's disease
MRI: Magnetic resonance imaging
FC: Fecal calprotectin
ESR: Erythrocyte sedimentation rate
CRP: C-reactive protein
NSAID: Nonsteroidal anti-inflammatory drugs
ROI: Region of interest
ROC: Receiver operating characteristic curves
ICC: Intraclass correlation coefficient
AUC: Area under the curve
IQR: Interquartile range
